# Central diabetes insipidus and pain medications – a risky combination

**DOI:** 10.1186/s40842-021-00124-9

**Published:** 2021-06-16

**Authors:** Teresa E. Pinto, Arati Mokashi, Elizabeth A. Cummings

**Affiliations:** Division of Endocrinology and Metabolism, Department of Pediatrics, Dalhousie University and IWK Health Centre, 5850/5980 University Avenue, Halifax, NS B3K6R8 Canada

**Keywords:** Diabetes insipidus, Opioid, Non-steroidal anti-inflammatory (NSAID), Hyponatremia

## Abstract

**Background:**

Central Diabetes Insipidus (CDI) results from decreased production of antidiuretic hormone (ADH) leading to an inability to concentrate urine. CDI is treated with desmopressin (DDAVP). Rarely reported in the literature, opioids and non-steroidal anti-inflammatories (NSAIDs) can induce hyponatremia in individuals treated for CDI.

**Case presentation:**

A 10-year-old boy with septo-optic dysplasia and CDI was treated with DDAVP 1.6 mg orally TID maintaining normal sodium levels. Post admission for a femur fracture, he was discharged on ibuprofen and hydromorphone. Sodium was 136 mmol/l two days before discharge.

He returned to the ED after having a seizure at home. He was euvolemic and mildly lethargic. Sodium was low at 108 mmol/l. DDAVP and hydromorphone were held and he was fluid restricted, but the sodium remained low. Sodium began to rise when Ibuprofen was stopped. Intermittent small doses of DDAVP were given to facilitate gradual correction of hyponatremia. At discharge, sodium had normalized.

**Conclusion:**

Hyponatremia has occasionally been described as a side effect of opioids and rarely of NSAIDs in patients with CDI. Stimulation of the thirst centre may play a role with opioids while a decrease in urine output may be the mechanism with NSAIDs.

**Supplementary Information:**

The online version contains supplementary material available at 10.1186/s40842-021-00124-9.

## Background

Central Diabetes Insipidus (CDI) is due to decreased or absent production of antidiuretic hormone (ADH). This results in an inability to concentrate urine and hypernatremia if access to sufficient free water is not possible. Desmopressin (DDAVP) is the mainstay of treatment. The Syndrome of Inappropriate ADH Secretion (SIADH), on the contrary, is the result of excessive ADH secretion. Clinically the patients are euvolemic with low serum sodium and osmolality and high urine sodium and osmolality with low urine output.

Opioids and non-steroidal anti-inflammatory drugs (NSAIDS) have both been reported individually, to cause hyponatremia, typically secondary to SIADH [1–3]. Rarely, have they been reported to cause hyponatremia in an individual with CDI [4, 5]. The mechanism for the hyponatremia would have to be different in this situation given that patients with DI do not produce ADH. To the best of our knowledge, hyponatremia with the use of NSAIDS or opioids in CDI has not been reported in the pediatric population nor has it been reported in patients treated with both concomitantly.

Here we report the case of a boy with CDI who presented with severe hyponatremia in the context of both opioid and NSAID treatment following a femur fracture. The patient’s mother provided written informed consent for the publication of this case.

## Case presentation

A 10-year-old boy with septo-optic dysplasia, adrenocorticotropic hormone (ACTH) and growth hormone (GH) deficiency, and CDI was treated with DDAVP 1.6 mg (tablets) orally four times daily maintaining normal sodium levels. CDI was diagnosed 3 years prior, when he presented with polyuria and polydipsia. A water depravation test showed high urine output after 20.5 h of fasting, a urine osmolality of only 462 mmol/kg, and a serum osmolality of 317 mmol/kg. He responded promptly to 10 µg of intranasal DDAVP with urine osmolality of 804 mmol/kg and a drop in urine output to less than 1 ml/kg/hour, confirming the diagnosis of CDI. He was also treated with hydrocortisone 6 mg orally twice daily (6.3 mg/m2/day) and growth hormone 1.3 mg by subcutaneous injection daily. Free T4 levels, monitored routinely, were normal with values of 10 pmol/l (8-22 pmol/l) 4 months prior to admission and 5 months post-admission. In addition, he was developmentally delayed with visual impairment and poor mobility and a body mass index of 34 kg/m2. Post-admission for a femur fracture, he was discharged on ibuprofen 600 mg q6hr prn and hydromorphone 1 mg q4hr prn which he was taking regularly. Sodium was 136 mmol/l (133–148) two days before discharge.

Less than 24 h later, he returned to the Emergency Department after having a seizure at home. His mother described it as an episode of increased tone in his arms, teeth grinding, and rocking after which he was unresponsive and apneic. She reported performing cardio-pulmonary resuscitation for 1 min before he started breathing again. Approximately 3 min later he regained alertness. In hospital, he was euvolemic and mildly lethargic. Blood pressure was 121/93 with a heart rate of 90–100. The patient had a brisk capillary refill, normal skin turgor and mucous membranes were noted to be moist. His glucose was 5.6 mmol/l however sodium was very low at 108 mmol/l. His mother denied any potential error in DDAVP administration and given his limited mobility he did not have free access to water outside of what his mother provided. She denied any additional water than his normal daily intake. She reported that the only change in his medications was the regular dosing of both ibuprofen and hydromorphone.

He was admitted to hospital and stress doses of hydrocortisone (15 mg three times daily) were initiated and continued for 3 days and double maintenance for 2 additional days until returning to maintenance doses. His hydromorphone was held and he was fluid restricted. Intermittent small doses of DDAVP were given initially to facilitate gradual correction of hyponatremia.

Sodium rose somewhat but remained below the normal range. Sodium normalized and remained stable when Ibuprofen was stopped (Table 1). At discharge, sodium had normalized on DDAVP 0.5 mg BID but he soon required higher doses again.

**Table 1:** Sodium Trend in Hospital

Day	Sodium (mmol/L)	Pain med (name/dose)	DDAVP (mg)
Prior to initial discharge	136	Hydromorphone 1 mg q4hr Ibuprofen 600 mg q4hrs	1.6 mg q6hr
1 of admission	108	Hydromorphone held	Held
2	110–118	No change	0.6 mg Prn
3	120–128	No change	0.6 mg Prn
4	127–137	No change	0.6 mg Prn
5	135–137	Ibuprofen held	0.6 mg Prn
6–14	137–141	None	0.5 mg BID, 0.1 mg prn
5 months post	139	None	1.4 mg qam, 0.6 mg qpm, 1.4 mg qhs

*Prn: as needed*

## Discussion

SIADH has been described as a potential side effect of opioids. Multiple potential mechanisms likely exist. Opioids may increase ADH secretion independently [6]. In addition, opioids block the reuptake of certain neurotransmitters, namely norepinephrine and serotonin. This in turn increases serotonin centrally. Serotonin increases ADH release centrally [7]. Finally, opioids work through opioid receptors (μ, ĸ, δ). Morphine binds to the μ receptor and has an inhibitory effect on the release of the neurotransmitter ƴ-aminobutyric acid (GABA) in the hypothalamic axis. Inhibition of GABA may result in an increase in ADH release [8], though the effects of GABA on vasopressin release show conflicting findings. Grossman suggests that opioids suppress ADH release suggesting that this mechanism is not the cause of hyponatremia [9]. Regardless, all potential mechanisms suggest an increase in ADH production or release as a cause of hyponatremia.

NSAIDs on the other hand, are felt to induce hyponatremia secondary to their potentiating effect on ADH action and resultant water retention. Renal prostaglandins typically inhibit ADH. Through decreasing renal prostaglandins, NSAIDs in turn result in increased ADH effect at the level of the kidney causing increased water retention and hyponatremia [6, 10]. Some have suggested that this occurs through prostaglandin E2’s (PGE2) inhibition of ADH-induced translocation of the aquaporin 2 receptor (AQP2) to the apical cell membrane of the collecting duct cells [11]. Ren and colleagues report that inhibition of PGE2 may actually increase ADH dependent translocation of the AQP2 receptor. Therefore the mechanism is unclear [12].

The current case is unique, however, as the patient had minimal endogenous ADH production. Bhat and colleagues reported on a 19-year-old woman with panhypopituitarism who developed hyponatremia after receiving hydrocodone for a wisdom tooth extraction. Interestingly, she experienced polydipsia postoperatively and then presented with hyponatremia. Subsequently, desmopressin was held after receiving morphine for an ambulatory ankle arthroscopic debridement and sodium remained in the normal range suggesting the cause of the original episode of hyponatremia was the use of opioids in association with DDAVP. No mention of increased thirst was made after the second surgery. The authors suggest that hydrocodone stimulated the patient’s thirst centre resulting in the hyponatremia [5]. Their hypothesis is supported by mouse models that show an increase in water intake with opiate agonists. It is proposed that the centre of action is the paraventricular nucleus of the hypothalamus which is rich in opioid receptors [13]. Our patient did not experience polydipsia post-operatively however, or at any time in the days following the surgery and water intake was closely monitored given his developmental delay, suggesting an alternative mechanism of action.

To our knowledge, there are only 2 cases in the literature that report hyponatremia in NSAID treated patients with DI. Bergoglio et al. described a 46-year-old man, taking desmopressin for CDI, who was admitted for symptomatic hyponatremia with a sodium of 113 mEq/L (113 mmol/L). The only change in his usual medication regime had been the addition of 200 mg/day of aceclofen for back pain [4]. The patient was reported to be euvolemic and had documented normal sodium levels prior to aceclofen. DDAVP was held and the patient was treated with hypertonic saline with resolution of the hyponatremia. Verrua et al. describe a 50-year-old man with known CDI who presented with a hyponatremic seizure and coma following 3 days of ketoprofen for cervical pain secondary to spondylosis. He was treated with 3% saline, and eventually, DDAVP was re-introduced. While he had likely taken NSAIDs before without symptomatic hyponatremia, the authors conclude that there may have been increased sensitivity to the effect due to age [14].

The current case is unique for several reasons. Firstly, it is the first case to our knowledge, that reports on a patient with CDI receiving both an opioid and an NSAID. It was noted that although the sodium improved with holding the opioid and fluid restriction, full recovery was not obtained until the NSAID was also held, suggesting a synergistic effect of both medications to cause hyponatremia. Secondly, the opioid effect would have to be mostly independent of ADH release given that this patient was known to have CDI requiring relatively high doses of DDAVP both before and after the event suggesting minimal endogenous ADH production. Thirdly, there was no evidence of increased water intake since his fluid intake was measured out due to his developmental delay and poor mobility and he was euvolemic at the time of presentation. The hyponatremia cannot, therefore, be explained by increased fluid intake. Finally, this is the first described case occurring in a pediatric patient. Adolescent brains have been shown to have a higher number of opioid μ-receptors than adult brains [15]. This might suggest a higher sensitivity to opioids in adolescents than adults which would suggest this may occur more commonly in youth. It may be under-recognized however, as the cause of hyponatremia in CDI may be misdiagnosed as DDAVP excess or excess fluid intake during times of illness.

This case brings to attention the importance of careful prescribing and monitoring when a patient with CDI requires pain medication. Review of this case led to quality improvement/patient initiatives at our centre. A complex care plan is filled out for such patients in order to bring awareness to other health care providers of potential risks associated with their condition (Additional file 1).

## Conclusion

Opioids and NSAIDs can both result in hyponatremia independently and together. This case suggests a synergistic effect that can be life-threatening. Cautious monitoring of sodium levels should take place whenever prescribing opioids or NSAIDs in this population with consideration of reducing or even temporarily discontinuing DDAVP while taking these medications. In addition, pharmacies should be aware and alert prescribers of this potential interaction while families and patients need to be educated in order to avoid potentially harmful outcomes.

## Supplementary Information

**Additional file 1:** Complex Care Plan.

## Data Availability

Not applicable.

## Abbreviations

CDI: Central Diabetes Insipidus
ADH: Anti-diuretic hormone
DDAVP: Desmopressin
NSAID: Non-steroidal anti-inflammatory
SIADH: Syndrome of inappropriate anti-diuretic hormone Secretion
ACTH: Adrenocorticotropic hormone
GH: Growth hormone
GABA: ƴ-Aminobutyric acid
PGE2: Prostaglandin E2
AQP2: Aquaporin 2
