# Supplementary material for: Central diabetes insipidus and pain medications – a risky combination
Source: Clin Diabetes Endocrinol. 2021 Jun 16;7:11. doi: 10.1186/s40842-021-00124-9 (PMC8207735; doi:10.1186/s40842-021-00124-9)
Supplement: Supplementary file 1 — Additional file 1: Complex Care Plan. [file 40842_2021_124_MOESM1_ESM.docx]

**Additional File 1: Complex Care Plan**


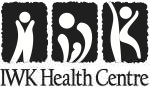
**Complex Care Management Plan**

**AFFIX PATIENT LABEL HERE**

K07002307 Jun/7/2002 M

SCA,TEST Visit

ER0000145/12 **HCN:** 22222222

Van den Hof, TEST / TEST, Maureen Dec/8/2012

- **Plan Developer of this record to notify admitting/emergency registration to enter VIP in MEDITECH**

This is a collaborative plan developed with involved professionals, patient and family.

Plan Date (dd/mm/yyyy): Prepared by:

The above named patient is known to have diabetes insipidus and is taking desmopressin (DDAVP) to control their fluid and electrolyte balance.

Failure to manage the patient appropriately can lead to life−threatening hypernatraemia or hyponatraemia.

Ensure free access to water. Allow patient to drink to their thirst.

If the patient is NPO, measure serum sodium (NA), determine last desmopressin dose, assess hydration status and call Endocrinology.

Never omit desmopressin unless clinically indicated or patient’s case is discussed with an Endocrinologist

If unable to take oral medication for any reason, parenteral (subcutaneous, IV or intranasal) desmopressin will be required.

Accurately monitor and record patient’s fluid intake and output.

Monitor patient’s serum sodium (Na) level (at least daily for inpatients) with the aim of keeping serum Na level within the normal range.

Contact an endocrinologist for urgent review or advice especially if using IV fluids, or sodium is abnormal.

Before starting any new medication, discuss with pharmacist and/or endocrinologist about potential drug interactions. This includes opioids and over the counter medications such as Ibuprofen or other non−steroidal anti−inflammatory medications, if used for more than 24 hours.

Choose acetaminophen as the preferred pain medication.

**MANAGEMENT OF Diabetes Insipidus**

**Contact Information:**

| **Signature / Status** | **Print Name** | **Initial** |
| --- | --- | --- |
|  |  |  |
|  |  |  |

■■■■■■■■■■■

■■■■■■■■■■■■■■

ER0000145/12

PERMANENT RECORD Page 1 of 1 06/16 IWKCOPAMA
